# Differential regulation of somatostatin receptors 1 and 2 mRNA and protein expression by tamoxifen and estradiol in breast cancer cells

**DOI:** 10.1186/1477-3163-4-10

**Published:** 2005-07-14

**Authors:** Juan A Rivera, Haydar Alturaihi, Ujendra Kumar

**Affiliations:** 1 Fraser Laboratories For Diabetes Research, Department of Medicine, Royal Victoria Hospital, McGill University Health Centre, Montreal, Quebec, H3A 1A1, Canada

**Keywords:** breast cancer cells, estradiol, cell proliferation, somatostatin receptors, tamoxifen

## Abstract

Somatostatin (SST) inhibition of hormone hypersecretion from tumors is mediated by somatostatin receptors (SSTRs). SSTRs also play an important role in controlling tumor growth through specific antiproliferative actions. These receptors are well expressed in numerous normal and tumor tissues and are susceptible to regulation by a variety of factors. Estradiol, a potent trophic and mitogenic hormone in its target tissues, is known to modulate the expression of SST and its receptors. Accordingly, in the present study, we determined the effects of tamoxifen, a selective estrogen receptor (ER) modulator (SERM), and estradiol on SSTR1 and SSTR2 expression at the mRNA and protein levels in ER-positive and -negative breast cancer cells. We found that SSTR1 was upregulated by tamoxifen in a dose-dependent manner but no effect was seen with estradiol. In contrast, SSTR2 was upregulated by both tamoxifen and estradiol. Combined treatment caused suppression of SSTR1 below control levels but had no significant effect on SSTR2. Treatment with SSTR1-specific agonist was significantly more effective in suppressing cell proliferation of cells pre-treated with tamoxifen. Taking these data into consideration, we suggest that tamoxifen and estradiol exert variable effects on SSTR1 and SSTR2 mRNA and protein expression and distributional pattern of the receptors. These changes are cell subtype-specific and affect the ability of SSTR agonists to inhibit cell proliferation.

## Introduction

Somatostatin (SST) is a regulatory neuropeptide produced and secreted by neuroendocrine and inflammatory cells [1]. It inhibits secretory and proliferative responses in a number of target cells. In tumors, SST not only blocks hormone hypersecretion but also causes variable degrees of tumor shrinkage. This antiproliferative effect involves cytostatic (growth arrest) and cytotoxic (apoptosis) actions. The biological effect of SST is mediated directly through a family of five specific high affinity G protein-coupled receptors termed SSTR1-5. These receptors are present in most normal and tumor cells [2]. SST also acts indirectly by reducing the synthesis and secretion of local and systemic growth promoting factors. Other actions of SST include inhibition of angiogenesis, promotion of vasoconstriction, and modulation of immune cell function [1,2].

Most solid tumors, including breast, prostate, colon, pancreas, brain and liver cancer, express variable levels of SSTRs and are, therefore, amenable to therapy with SST analogues [1-3]. The anti-proliferative effects of SST and its analogue octreotide (OCT) in breast and other cancers have been clearly demonstrated [4-8]. In animal models of breast cancer, OCT enhanced the anti-neoplastic effect of tamoxifen (Tam) or castration [6]. In addition, SST analogues cause a potent reduction in circulating levels of growth hormone (GH) and its mediator hormone IGF-1, a potent mitogen in breast and other cancers [9]. Clinical studies, however, have failed to demonstrate a clinically significant benefit of OCT administration in addition to standard therapy with tamoxifen in patients with advanced breast cancer [10,11]. Although most tumors in patients with primary breast cancer express SSTRs [12], lower expression levels in more aggressive tumors may account for the failure of OCT with Tam in these settings [13]. Therefore, upregulation of SSTR expression in aggressive tumors may be a desirable therapeutic goal.

Some of the factors known to upregulate SSTR expression are also cell proliferation promoters. This presumably works as a way of endogenous counter regulation [1]. In several tissues, the sex hormone estradiol (E2) acts as a potent proliferative and mitogenic factor and is also known to increase the expression of SST and its receptors [14-18]. Previous studies have described the variable effects of E2 on SSTR-mRNA in a number of cell lines [14-18]. In breast cancer cells, treatment with E2 caused up-regulated SSTR2-mRNA expression, while Tam had variable effects depending on the cell line used [14,15]. Visser-Wisselaar *et al *[16] reported induction of SSTR2 and SSTR3 expression by estrogen in transplantable rat prolactin-secreting pituitary tumor cells. Similarly, Djordijevic *et al *[17] reported positive regulation of SSTR2 and SSTR3 alongside inhibition of SSTR1-mRNA by E2 in primary cultures of female rat pituitary cells expressing all five SSTRs. In contrast, Kimura *et al *[18] showed upregulation of SSTR1, SSTR2, and SSTR3, and drastic downregulation of SSTR5 in pituitary cells of ovariectomized rats treated with E2 for one month. From these studies, it is clear that the precise regulatory role of estrogens on SSTRs and its implications in cell growth control need further elucidation. Additionally, the role of Tam has not been investigated in detail.

Tam, an ER modulator, potently inhibits the growth of ER-positive breast cancer cells [19]. Cancer cells from tissues not classically considered estrogen-dependent (e.g. thyroid, skin, pancreas, liver, glia and meninges) also show an inhibitory proliferative response to Tam [20-24]. Some of the growth inhibitory properties of Tam are related to its ability to modify the expression of cell growth regulators. One such regulator, transforming growth factor β1 (TGF-β1), an inhibitor of cell proliferation, is upregulated by Tam in both ER-positive and ER-negative cells [23,25]. In contrast, Tam reduces circulating levels of IGF-1 [26] and interferes with the IGF-1 receptor signalling pathway in breast cancer cells by reducing its phosphorylation and by inhibiting the induction of its substrate IRS-1 [27,28].

It has been previously shown that SSTR1 and SSTR2 are highly expressed in breast cancer cells [29] and exert an inhibitory role on tumor cell proliferation and migration [30-35]. Accordingly, in the present study, we compare the effect of Tam and E2, alone or in combination, on SSTR1 and 2 expressions in breast cancer cells at the mRNA and protein levels. We report that Tam and E2 exhibited contrasting effects on SSTR1 in ER-positive cells. However, their actions on SSTR2 expression in ER-negative cells were similar. We further discuss our findings in terms of the potential clinical implications of such interactions.

## Materials and methods

### Reagents

All the culture cells were obtained from ATCC. Tamoxifen, Estradiol and 3-(4, 5-dimethylthiazolyl-2)-2, 5-diphenyltetrazolium bromide (MTT) were purchased from SIGMA (Sigma-Aldrich Canada Ltd). Anti rabbit fluorescein isothiocyanate (FITC)-conjugated secondary antibodies were obtained from Jackson Laboratory. Cell culture medium was from Invitrogen and FBS was purchased from Wisent, Canada. All other reagents were purchased from various suppliers as indicated.

### Cell lines

ZR-75-1 and T47D cells were maintained in RPMI Medium 1640 (Gibco BRL) at standard conditions (humidified atmosphere, at 37°C, with 5% CO_2_). MDA-MB-231 cells were maintained in Leibovitz's L-15 medium at 37°C. Culture media were supplemented with 10% fetal bovine serum (FBS) and standard antifungal-antibacterial treatment. Culture media were replaced 24 hours prior to treatment with phenol red-free media supplemented with 10% dextran-coated charcoal-treated FBS. Experiments were conducted on cells between passages 4 to 8.

### Cell Treatment

At ~70% confluency, cells were treated with increasing concentrations (10^-10 ^M, 10^-8 ^M and 10^-6 ^M) of Tam and β-estradiol (E2), alone or in combination. Time course experiments (6, 12, 24, 48, and 96 h) showed that changes in mRNA levels were already maximal at 24 h and sustained thereafter. Thus a 24–36 h exposure time was used for these experiments. In order to determine the expression of SSTR1 and SSTR2 by immunocytochemistry, cells were treated with 10^-6 ^M of the corresponding compound for 30–36 h. For cell proliferation experiments, cells were exposed to Tam, E2, both or none for 30 h. 0.5 μM of the nonpeptide SSTR1-specific agonist L-797, 591 [36] or vehicle was added to the medium, and proliferation rate were assessed after an additional 42 h.

### RT-PCR

Total RNA was extracted using TRIzol Reagent (Invitrogen) according to the manufacturer's instructions. Potential contaminating genomic DNA was degraded by incubation with RQ1 ribonuclease-free deoxyribonuclease (Promega Corp.) for 30 minutes at 37°C in the presence of RNAse inhibitor (Invitrogen). 10 μg of total RNA was reverse transcribed (RT) using M-MLV-reverse transcriptase (Life technologies Inc), and then amplified using specific primers for SSTR1, SSTR2 and β-actin as an internal control. Primers used were as follows:

SSTR1-forward 5' TATCTGCCTGTGCTACGTGC 3' (nt 714–733)

SSTR1-reverse 5' GATGACCGACAGCTGACTCA 3' (nt 911–930)

SSTR2-forward 5' ATCTGGGGCTTGGTACACAG 3' (nt 600–619)

SSTR2-reverse 5' CTTCTTCCTCTTAGAGGAGC 3' (nt 728–747)

β-actin-forward 5' ATCATGAA GTGTGACGTGGAC 3' (nt 885–905)

β-actin-reverse 5' AACCGACTGCTGTCA CCTTCA 3' (nt 1325–1345)

For PCR amplification 4 μl of RT products (cDNA) were mixed in 100 μl total volume of PCR-buffer (Invitrogen) containing 2% DMSO, 2.25 mM MgCl_2_, 50 μM of each dNTP, 15 pmol of SSTR-primers, and 3 pmol of β-actin primers.

After initial denaturation at 94°C for 10 min, *Taq *DNA polymerase (Invitrogen) (2.5 U/reaction) was added and samples were subjected to 38 cycles of denaturation at 94°C for 1 min, annealing at 58°C for 45 sec, and extension at 72°C for 45 sec, followed by a 10 min final extension at 72°C. 20 μl of the PCR products were fractioned by electrophoresis in 1.5% agarose gels containing ethidium bromide and visualized under UV light. The specificity of the amplified SSTR products and the corresponding observed bands were confirmed by Southern blot hybridization using specific ^32^P-dCTP-random primer-labelled SSTR cDNAs. AlphaEaseFC (Alpha Innotech Corporation, San Leandro, CA) was used for optical density (OD) measurements of the product. OD of target band was corrected for the corresponding β-actin-band (OD ratio) and then normalized against the OD ratio of non-treated cells (OD index).

### Immunocytochemistry

Expression of SSTRs in ZR-75-1, T47D and MDA-MB-231 were determined by immunocytochemistry using rabbit polyclonal antibodies for hSSTR1 and hSSTR2, diluted as previously described [37,38]. Briefly, cells were grown in 24-well plates to 60% confluency, and then treated with Tam or E2, alone or in combinations. Cells were fixed with 4% paraformaldehyde and incubated overnight at 4°C with primary antibodies diluted (1:300) in PBS. After three subsequent washes in PBS, cells were incubated at room temperature for 1 h with FITC-conjugated secondary antibodies (goat anti-rabbit IgG). The specificity of immunofluorescence was determined in the absence of hSSTR-specific antibodies.

### Cell Proliferation Assay

Cell proliferation was assessed 72 h post-treatment by the MTT method. Cells were seeded at 1000 cells/well in 96-well plates containing 100 μl of standard medium. Treatments were started, as described above, 24 h later. At the end of the treatment period, 25 μl MTT solution, 5 mg/ml in PBS was added to each well. Following 2 h incubation at 37°C, 100 μl stop solution (50% dimethyl-formamide, 50% H_2_O, 20% SDS, pH 4.7) was added to each well and cells were incubated for an additional 20 h to solubilize the crystallized dye. Optical densities of the solutions, in each well, were determined by spectrophotometer.

### Statistical Analysis

Results are expressed as means ± SE. Statistical significance was determined using the Student's unpaired t test.

## Results

### Effects of Tamoxifen and Estradiol on SSTR1 and SSTR2 mRNA and Protein Expression in ZR-75-1 Cells

**Figure 1:**
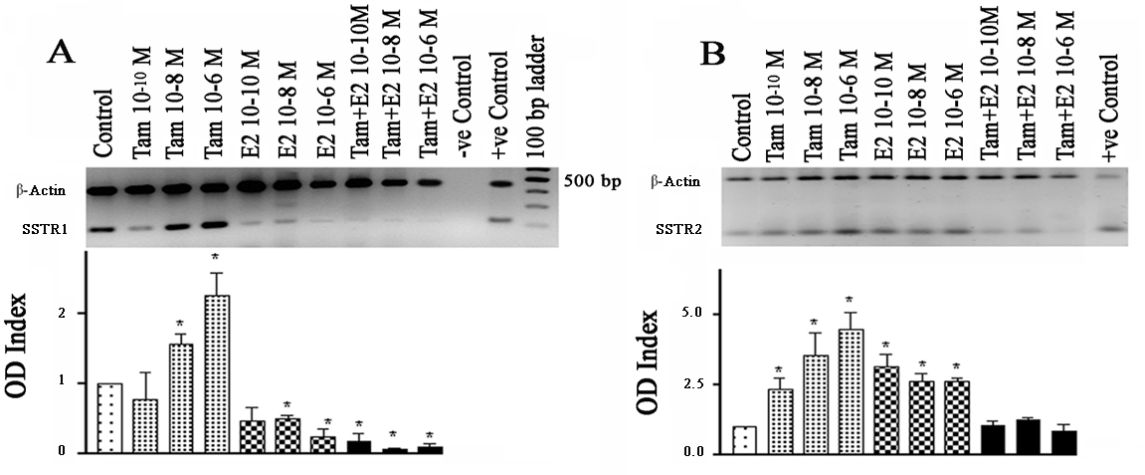
Concentration dependent changes in SSTR1 (A) and 2 (B) mRNA in ZR-75-1 cells treated with Tam and E2 alone or in combination. Cells were treated with Tam and E2 for 24 h (see Materials and Methods for details). mRNA levels were determined by RT-PCR and expressed as OD ratio. Note a concentration-dependent increase in SSTR1 mRNA levels with Tam and decreased expression in the presence of E2 or combined treatment. SSTR2 mRNA levels increased in response to Tam or E2 but not with combined treatment. Data presented are from three independent experiments performed in triplicate. A representative ethidium bromide-stained gel image is shown. * p < 0.05

In order to assess the effects of Tam and E2 on SSTR1 and SSTR2 expression, we first determined dose-dependent changes in mRNA expression. In the ER-positive breast cancer cell line ZR-75-1, Tam treatment resulted in a significant increase in SSTR1-mRNA, up to 2.2-fold in comparison to non-treated cells, at high concentrations (10 nM and 1 μM) (Fig. 1A). In contrast, E2 treatment significantly decreased SSTR1-mRNA expression by >25%. Treatment with both Tam and E2 resulted in a further decrease in SSTR1 mRNA levels. In contrast, both Tam and E2 increased SSTR2 mRNA expression. However, the effect of Tam was stronger (4.5 fold increase at 1 μM) in addition to being dose-dependent (Fig. 1B). In this case, simultaneous treatment with Tam and E2 had no significant effect on SSTR2 mRNA expression as compared with control (Fig. 1B).

**Figure 2:**
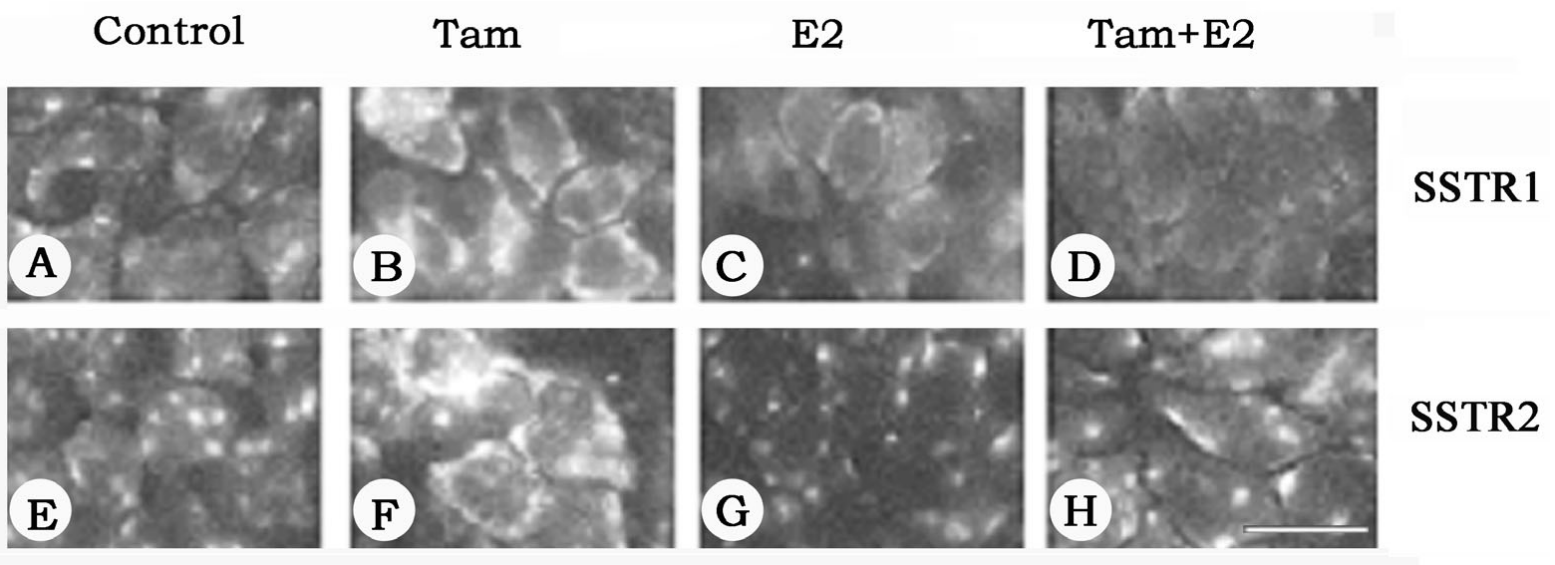
Photomicrographs illustrating the immunohistochemical localization of SSTR1 and 2 in ZR-75-1 breast cancer cells. Cells were treated with 1 μM of Tam and E2, alone or in combination, for 30–36 h and labelled with anti-rabbit SSTR1 and 2 antibodies followed by FITC-conjugated goat anti-rabbit IgG. Note a significant increase in SSTR1 and 2-like immunoreactivity in response to Tam (B and F) and decreased staining in the presence of E2 (C and G). Upon combined treatment SSTR1 is less than control (D) but SSTR2 expression is comparable to the control (H). Scale bar = 25 μM.

We further extended our study and determined the cellular distribution of SSTR1 and SSTR2 by indirect immunofluorescence using SSTR1- and SSTR2-specific antibodies. As shown in Fig. 2, in ZR-75-1 cells, the changes observed after a 30–36 h treatment were comparable to changes described at the mRNA level. In control cells, SSTR1 and SSTR2 immunoreactivity had patchy distributions on the cell surface. Upon Tam treatment, SSTR1 and SSTR2 immunoreactivity significantly increased and the receptors were more uniformly expressed on the cell surface (Fig. 2B and 2F). Treatment with E2 alone or in combination with Tam resulted in either decreased expression or no significant changes in SSTR1- and SSTR2-like immunoreactivity at all time-points (Fig. 2C,D,G and 2H).

### Effects of Tamoxifen and Estradiol on SSTR1 and SSTR2 mRNA and Protein Expression in T47D Cells

**Figure 3:**
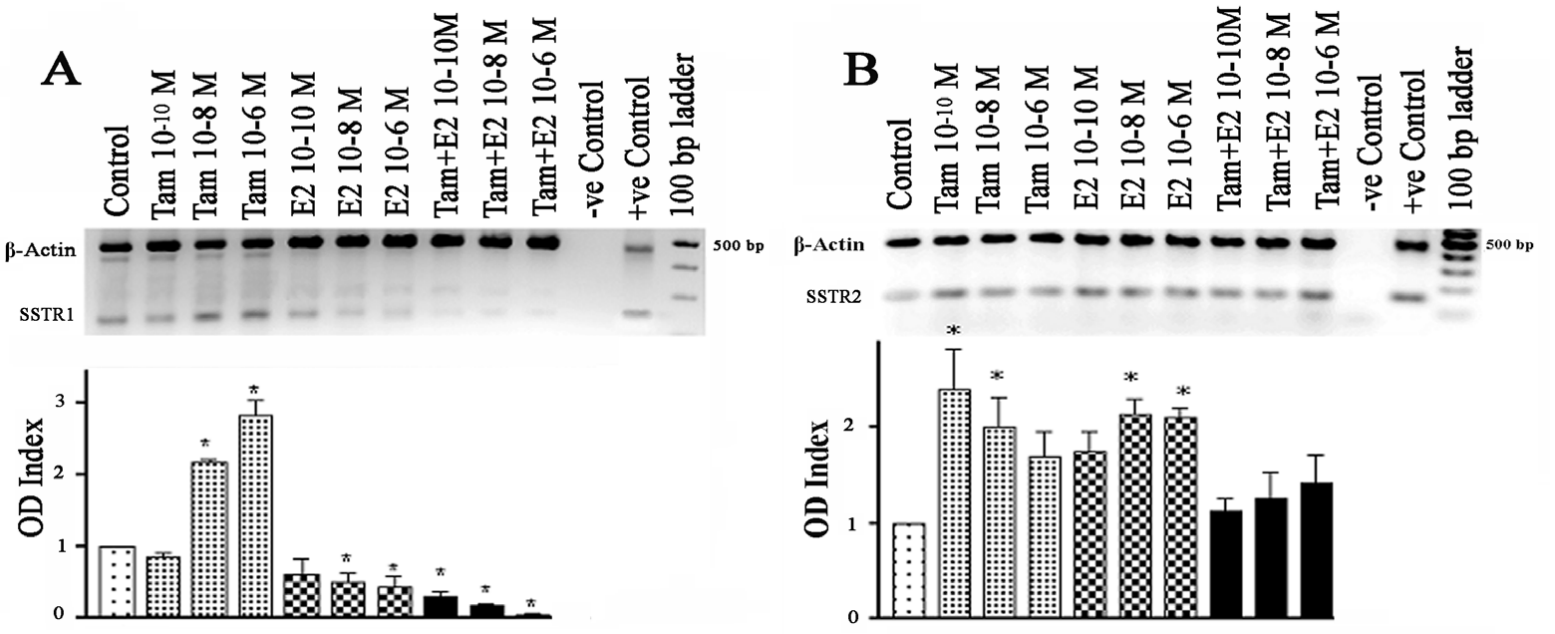
Semiquantitative analysis of SSTR1 (A) and SSTR2 (B) mRNA in T47D cells treated with Tam and E2, alone or in combination, for 24 h. mRNA levels were estimated by RT-PCR and expressed as OD ratio. SSTR1 gene expression increased in the presence of Tam but decreased with E2 and combined treatments in a concentration-dependent manner. SSTR2 gene expression increased in the presence of Tam as well as with E2 but not with the combined treatment. A representative ethidium bromide-stained gel image is shown. Data presented are from three independent experiments performed in triplicate. * p < 0.05.

**Figure 4:**
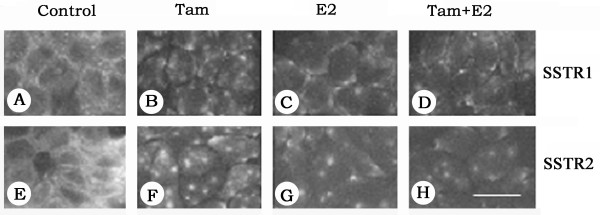
Photomicrographs depicting the immunohistochemical localization of SSTR1 and 2 in T47D breast cancer cells. Cells were treated with 1 μM Tam and E2, alone or in combination, for 30–36 h and processed for localization of SSTR1 and SSTR2 as described in Fig. 2. Note the significant changes in distributional pattern of SSTR 1 (A–D) and 2 (E–H) like immunoreactivity upon treatment. In control cells, both receptors are uniformly expressed as membrane proteins; however, upon treatment, a punctated receptor-like immunoreactivity was noticed at the cell surface. Scale bar = 25 μM.

To assess whether these effects were similar in other ER-positive cell lines, we studied T47D cells. Under estrogen-free conditions, non-treated T47D cells express mRNA for both SSTR1 and SSTR2 (Fig. 3A and 3B). SSTR1 mRNA exhibited a biphasic response to Tam treatment, slightly decreasing with 0.1 nM Tam while increasing by up to ~300% with 1 μM (Fig. 3A). In contrast, E2 down regulated SSTR1 mRNA. Combined treatment caused a greater decrease in SSTR1 mRNA levels compared to E2-treated cells. In comparison, SSTR2-mRNA in T47D cells increased with Tam or E2. However, the combined treatment did not significantly change SSTR2 mRNA expression compared to control cells (Fig. 3B). In order to examine the cellular distribution of SSTR1 and 2 in response to Tam and E2, alone or in combination, on receptor expression at the protein level, T47D cells were also studied by indirect immunofluorescence. In control cells (Fig. 4), both receptors are expressed as membrane proteins. SSTR1 and 2, immunoreactivity decreased in T47D cells upon 30–36 h treatment with either Tam or E2, alone or in combination. Furthermore, a punctate pattern of receptor immunoreactivity was detected in all treated cells (Fig. 4).

### Effects of Tamoxifen and Estradiol on SSTR1 and SSTR2 Expression in MDA-MB-231 Breast Cancer Cells

**Figure 5:**
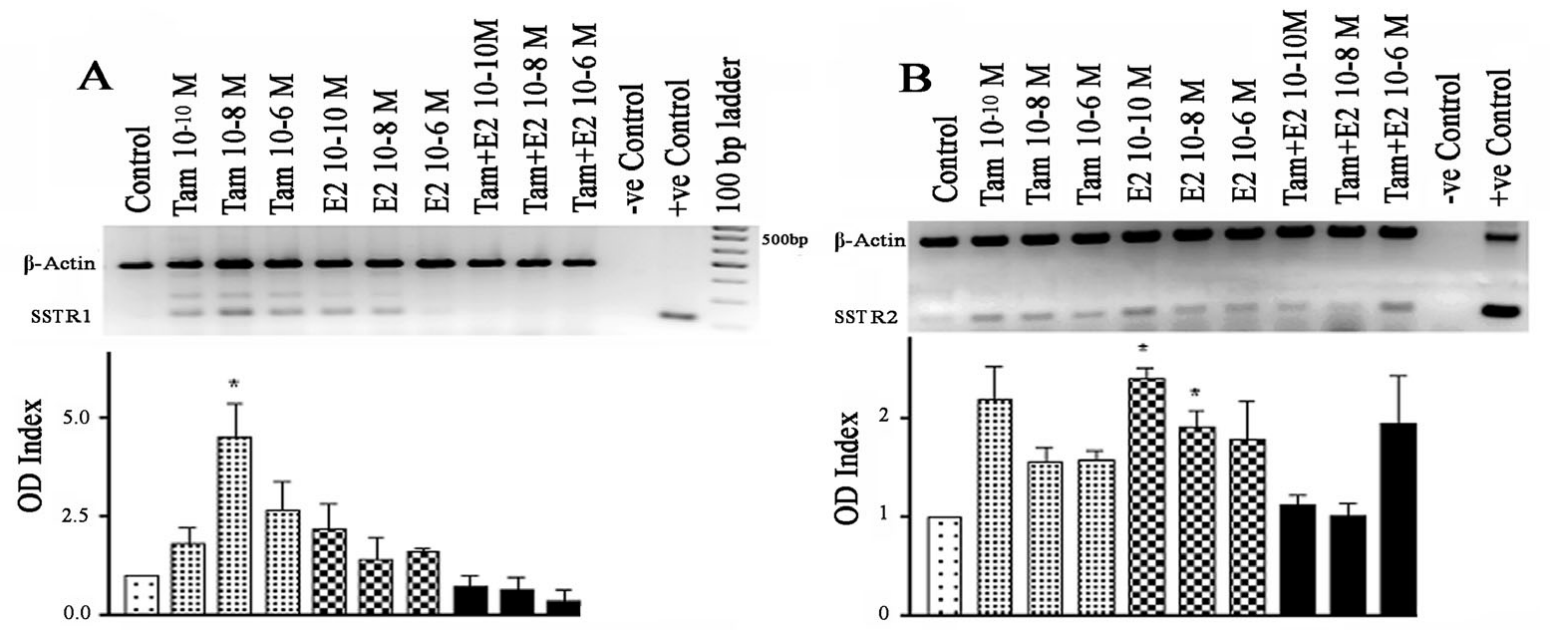
Concentration-dependent changes in SSTR1 and 2 mRNA in MD-MB231 cells treated with Tam or E2, alone or in combination, for 24 h. mRNA levels were determined by RT-PCR and expressed as OD ratio. No significant changes in receptor expression were seen except for SSTR1 in presence of 10 nM of Tam and SSTR2 at the lower concentration of E2. Data presented are from three independent experiments performed in triplicate. A representative ethidium bromide-stained gel image is shown. * p < 0.05.

We next examined the effects of Tam and E2 in ERα-negative breast cancer cells MDA-MB-231. At baseline conditions, cells appeared to express a low amount of SSTR1-mRNA in comparison to SSTR2 (Fig. 5). Tam treatment significantly increased SSTR1-mRNA at a 10 nM concentration (Fig. 5A). The increase observed in SSTR2 with Tam was modest and did not reach statistical significance (Fig. 5B). Similar changes occurred when cells were treated with E2: both SSTR1 and SSTR2-mRNA were upregulated; however, in this case, the effect on SSTR2 was stronger. The combined treatment resulted in a trend towards down-regulation of SSTR1-mRNA while there was no significant change in SSTR2-mRNA compared to control.

**Figure 6:**
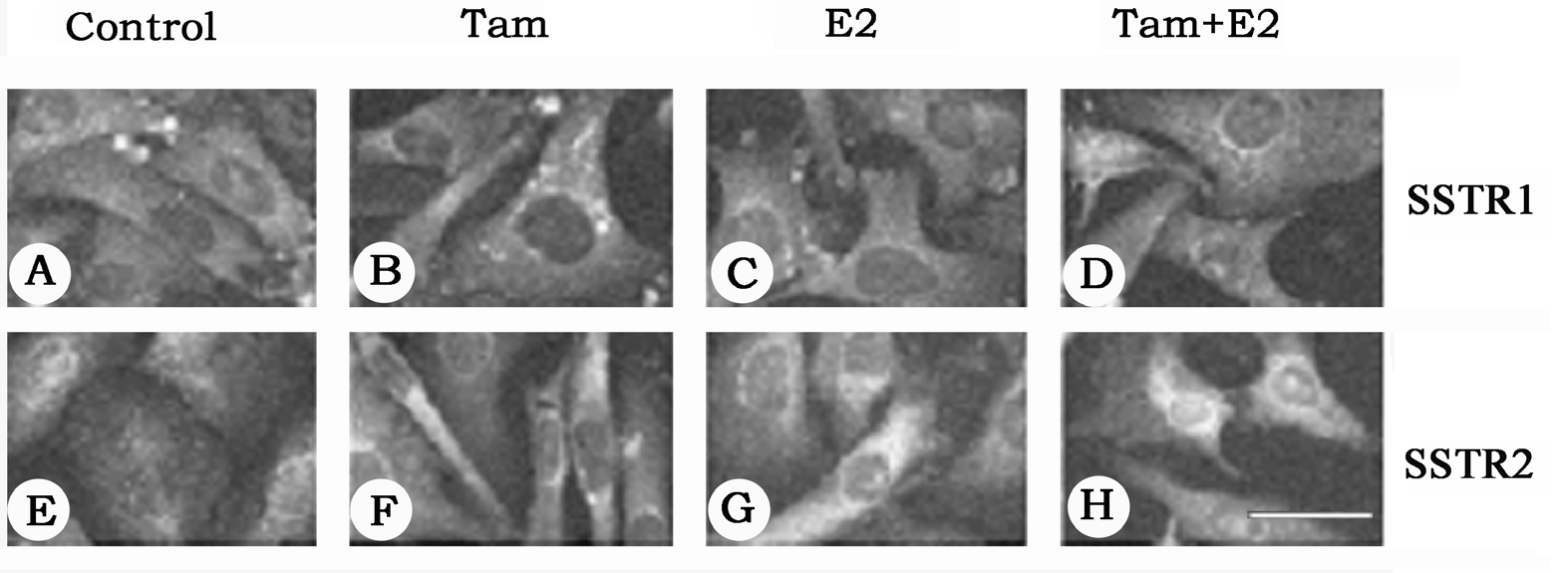
Photomicrographs illustrating the immunohistochemical localization of SSTR1 (A–D) and SSTR2 (E–H) in the ERα negative MDA-MB-231 breast cancer cells. Cells were treated with 1 μM Tam and E2, alone or in combination, for 30–36 h and processed for localization of SSTR1 and SSTR2 (see Fig. 2 for details). MDA-MB-231 cells exhibited weak expression of both receptors. Note the apparent intracellular localization of the receptors like immunoreactivity. Scale bar = 25 μM.

Similarly, very mild SSTR1 and SSTR2 immunoreactivity was observed in MDA-MB-231 cells under basal conditions (Fig. 6). The distribution followed a diffuse punctate pattern for both receptors. SSTR1 and SSTR2 were upregulated after Tam treatment while SSTR2 immunoreactivity was also upregulated by E2. Combined treatment was also associated with modest increases in SSTR1 and SSTR2 immunoreactivity.

### SSTR1 Selective Agonist Accelerates the Tamoxifen-Induced Inhibition of ZR-75-1 Cell Proliferation

**Figure 7:**
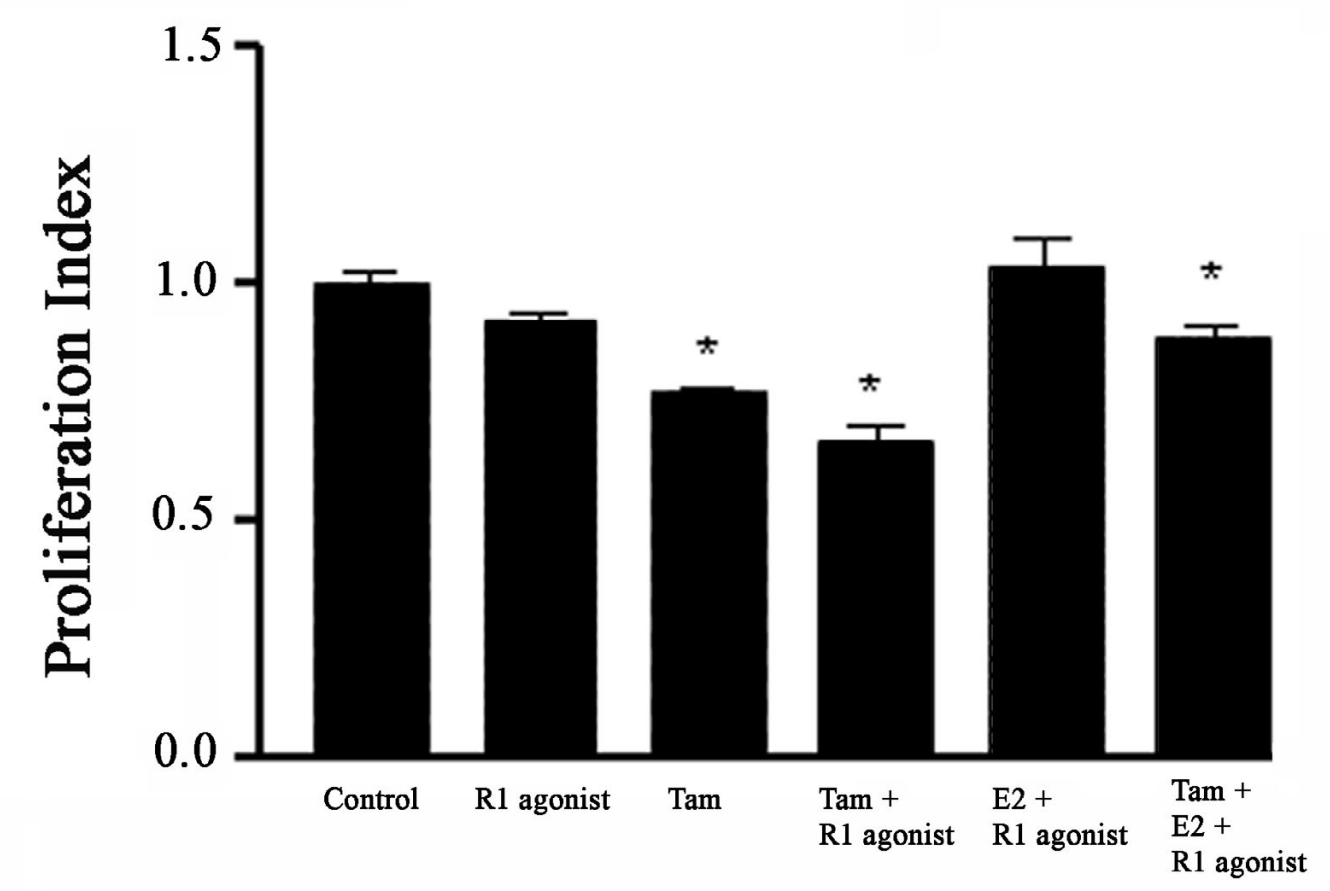
SSTR1 selective agonist enhanced the antiproliferative effects of Tam in ZR-75-1 cells. Cells were pre-treated with Tam or E2 for 30 h. after the initial 30 h. Subsequently, a non-peptide SSTR1-specific agonist (R1 agonist) was added, as indicated, and cells were cultured for an additional 42 h. Cell proliferation was determined by MTT assay as described in Material and Methods. Data are presented as proliferation index in comparison to control as an arbitrary unit.

As illustrated in Fig. 7, the SSTR1-specific agonist potentiated the antiproliferative effects of Tam in ZR-75-1 cells. Cells were pretreated with Tam and E2, alone or in combination, for 30 h and then exposed to SSTR1-selective agonist for 42 h. Upon treatment with Tam alone, a significant reduction in cell proliferation was observed in ER-positive breast cancer cells. Combining SSTR1-specific agonist with Tam further suppressed proliferation when compared with Tam alone. It is worth noting that treatment with SSTR1 agonist alone had no significant effect on cell proliferation. Interestingly, when both Tam and E2 were given simultaneously followed by SSTR1 agonist, there was a mild but significant decrease in cell proliferation rate. Furthermore, in T47D and MDA-MB-231 cells, pretreatment with Tam followed by SSTR1-specific agonist resulted in mild, yet non-significant, additional inhibition of proliferation compared with Tam alone (data not shown).

## Discussion

In the present study, we demonstrate the roles of Tam and E2 on SSTR1 and SSTR2 mRNA and protein expression in ER-positive and ER-negative cells. This is the first study that not only systematically analyses the regulation of SSTR by E2 and Tam in breast cancer cells at both the mRNA and protein levels, but also demonstrates how this translates into changes in cancer cell proliferation control with somatostatin analogs. Our first observation was the contrasting effects of E2 and Tam on SSTR1 in ER-positive cells. An inhibitory effect of E2 on SSTR1-mRNA has been previously reported in rat anterior pituitary cells [17]. SSTR1 has been shown to inhibit cell proliferation and migration of tumor cells [30-33]. Therefore, it is most likely that its down-regulation by E2 plays a role in the proliferative effect of E2 in susceptible tissues. Likewise, the observed up-regulation of SSTR1 by Tam may represent an important mechanism whereby Tam exerts an inhibitory effect on tumor progression. In CCL39 human fibroblasts expressing SSTR1, SST inhibited activation of Rho (a key regulator of the actin-based cell cytoskeleton), thereby inhibiting the assembly of focal adhesions and actin stress fibres, and impairing cell migration [30]. This effect was not seen in cells expressing only SSTR2. Moreover, SSTR1-expressing cells from GH- and PRL-secreting adenomas and medullary thyroid cancer exhibited inhibition of hormone secretion in addition to reduced cell viability in response to SSTR1-specific agonist [31,32].

SSTR2 mRNA up-regulation by both Tam and E2 is in agreement with previous studies in T47D cells but differs from what has been previously shown in ZR-75-1 and MDA-MB-231 cells [14,15]. Xu *et al *[14] observed no effect of E2 in MDA-MB-231 cells, while we found that these cells responded to nanomolar concentrations of Tam and E2 with upregulation of SSTR1 and SSTR2 mRNA. This discrepancy can be explained by the different methods used: nuclease protection assay by Xu *et al*, and RT-PCR by us, the latter being more sensitive to small changes. Furthermore, Xu *et al *showed in ZR-75-1 cells that only E2 caused SSTR2 upregulation while Tam opposed this effect. It has been shown that exposure to E2 can increase ERβ mRNA expression in breast cancer cells [39]; conversely, prolonged E2 deprivation can induce loss of ER expression in some breast cancer cell lines [40]. Therefore, we speculate that differences in the E2-free pretreatment period (48 h in Xu *et al *vs. 24 h in our experiments) could have altered ER expression at the time of treatment. Additionally, the use of Tam in our experiments vs. the active metabolite OH-tamoxifen (a product of liver metabolism of Tam *in vivo*) in the experiment of Xu *et al *may be responsible for these differences.

Given the fact that in ERα-negative cells, E2 and Tam displayed similar effects, our data provide evidence that the upregulation of SSTR2-mRNA by ER agonists is, at least in part, independent of ERα. Contrary to previous beliefs, the classical ER-negative breast cancer cell line MDA-MB-231 expresses low levels of ERβ but no ERα [39]. Kimura *et al *[41] have recently shown estrogen sensitive sequences in the promoter region of SSTR2 gene. Further studies are required to delineate the exact molecular mechanisms involved. Given that the affinity of Tam for the ER is lower than that of its metabolite OH-tamoxifen or E2 [42], it is not surprising that simultaneous treatment with equimolar concentrations of E2 and Tam resulted in down-regulation of SSTR1 as seen when E2 was given alone. It is remarkable; however, that the effect was stronger with the combined treatment and that, in the case of SSTR2, the combined E2 and Tam treatment resulted in loss of the upregulatory effect of either compound alone. These effects probably involve interactions between ERs and their coregulators, and may include E2 upregulation of ERβ expression and induction of the formation of ERα/ERβ heterodimers with decreased transcriptional activity [43,44].

Of interest, we detected surprising changes in the pattern of subcellular distribution of the receptors as a result of the treatments used. Basically, Tam changed the pattern of expression of SSTR1 and SSTR2 in ZR-75-1 cells from patchy to a more homogeneous cell surface distribution. In contrast, both Tam and E2 altered the homogeneous distribution of receptors in T47D cells to a more irregular patchy distribution. These changes in SSTR1 and SSTR2 immunoreactivity may involve receptor homo- or heterodimerization, receptor complex dissociation and/or internalization [45,46]. These could be a specific effect of ER activation or the result of changes in the fluidity and/or composition of the cell membrane. The ability of Tam to affect membrane stability by decreasing its fluidity has been previously shown. There are reports of Tam decreasing synthesis of glycosphingolipids by blocking synthesis of their precursor, glycosylceramide, thereby causing intracellular ceramide accumulation and membrane disruption [47].

In summary, our data show that SSTR1 and SSTR2 expression in breast cancer cells is modulated by Tam and E2 in a cell-specific manner. SSTR1 expression is upregulated by Tam while E2 and combined treatment preferentially downregulate this receptor in ER-positive cells. In ERα-negative cells, both ligands caused upregulation of SSTR1. The changes in subcellular distribution of SSTR1 and SSTR2 were favourable in only one of the ER-positive cell lines investigated (ZR-75-1). These cells also showed a stronger growth inhibitory response to SSTR1-agonist following pretreatment with Tam. Therefore, SSTR1 activation appears to be a potentially important mechanism for tumor cell growth control. For this reason, enhancing SSTR expression in tumor cells could make them more susceptible to growth inhibition by SSTR agonists, particularly SSTR1 agonists, as anti-neoplastic treatment. Our study suggests that SERMs like Tam may have such effects. These results have important implications in our understanding of the role of SSTRs in ER responsiveness of breast cancer. Moreover, it might not be premature to anticipate that, based on our results, some breast cancer patients may benefit from SSTR1-selective agonist therapy in combination with Tam, in an estrogen-depleted milieu.

## Abbreviations

E2, estradiol; ER, estrogen receptor; FBS, fetal bovine serum; GH, growth hormone; IGF-1, insulin-like growth factor 1; IRS, insulin responsive substrate 1; mRNA, messenger ribonucleic acid; MTT, 3-(4,5-dimethlthiazolyl-2)-2, 5-diphenyltetrazolium bromide; RT-PCR, reverse transcriptase polymerase chain reaction; SERM, selective estrogen receptor modulator; SST, somatostatin; SSTR, SST receptor; Tam, tamoxifen; TGF, transforming growth factor; OD, optical density.
